# Colonization Potential to Reconstitute a Microbe Community in Pseudo Germ-Free Mice After Fecal Microbe Transplant From Equol Producer

**DOI:** 10.3389/fmicb.2020.01221

**Published:** 2020-06-05

**Authors:** Wenou Liang, Lichao Zhao, Jingfeng Zhang, Xiang Fang, Qingping Zhong, Zhenlin Liao, Jie Wang, Yingyu Guo, Huijun Liang, Li Wang

**Affiliations:** ^1^College of Food Science, South China Agricultural University, Guangzhou, China; ^2^Guangdong Provincial Key Laboratory of Nutraceuticals and Functional Foods, College of Food Science, South China Agricultural University, Guangzhou, China

**Keywords:** pseudo germ-free mice, fecal microbiota transplantation, equol producer, human microbiota-associated mice model, 16S rRNA gene amplicon sequencing

## Abstract

Human intestinal microbiota plays a crucial role in the conversion of isoflavones into equol. Usually, human microbiota-associated (HMA) animal models are used, since it is difficult to establish the mechanism and causal relationship between equol and microbiota in human studies. Currently, several groups have successfully established HMA animal models that produce equol through germ-free mice or rats; however, the HMA model of producing equol through pseudo germ-free mice has not been established. The objective of this study is to establish an HMA mice model for equol production through pseudo germ-free mice, mimicking the gut microbiota of an adult human equol producer. First, a higher female equol producer was screened as a donor from 15 volunteers. Then, mice were exposed to vancomycin, neomycin sulfate, metronidazole, and ampicillin for 3 weeks to obtain pseudo germ-free mice. Finally, pseudo germ-free mice were inoculated with fecal microbiota of the equol producer for 3 weeks to establish HMA mice of producing equol. The results showed that (i) the ability to produce equol was partially transferred from the donor to the HMA mice. (ii) Most of the original intestinal microbiota of mice were eliminated after broad-spectrum antibiotic administration. (iii) The taxonomy data from HMA mice revealed similar taxa to the donor sample, and the species richness returned to the level close to the donor. (iv) The family *Coriobacteriaceae* and genera *Collinsella* were successfully transferred from the donor to HMA mice. In conclusion, the HMA mice model for equol production, based on pseudo germ-free mice, can replace the model established by germ-free mice. The model also provides a basis for studying microbiota during the conversion from isoflavones into equol.

## Introduction

Isoflavones are polyphenolic substances found in soy and related products. They are known to possess high estrogenic activity (Zhou et al., 2019). Isoflavones are of significant interest for treatment of osteoporosis, cardiovascular disease and cancer, since they match the chemical structure of estrogens and bind to human estrogen receptors (Yang et al., 2016; Rienks et al., 2017). These functions are often determined by the gut active metabolites of isoflavones (Setchell and Clerici, 2010). In nature, isoflavones are primarily encountered as conjugates (>80%) of sugars, in the form of isoflavone-glycosides (daidzin, genistin, and glycitin). These compounds are not easily available, and also have low bioactivity. Aglycones (daidzein, genistein, and glycitein) need to be released from their glycosides before achieving full activity, due to the action of glycosyl hydrolases (members of the β-glucosidase family). The formed aglycones are finally transformed to the more biologically active equol by the action of reductases (daidzein reductase, dihydrodaidzein reductase, tetrahydrodaidzein reductase, and dihydrodaidzein racemase) (Guadamuro et al., 2015). It has been suggested that intestinal microorganisms, capable of producing equol enzymes, are responsible for the transformation of isoflavones into equol. The majority of the equol producing bacteria, characterized so far, are part of the family *Coriobacteriaceae*, such as *Adlercreutzia equolifaciens* DSM 19450^T^, *Slackia isoflavoniconvertens* DSM 22006, *Slackia equolifaciens* JCM 16059^T^ (Maruo et al., 2008; Jin et al., 2010; Shimada et al., 2012). However, our current knowledge on equol production is still limited, and there are insufficient research data to show that this family is the only intestinal group acting on isoflavones and producing equol. Although scientific information about gut microbes, their enzymes, and related pathways are accruing, our current knowledge is still limited.

The human microbiota-associated (HMA) animal models are viable alternatives to human studies, since it is challenging to establish the mechanism and causal relationship between equol and microbiota, due to sampling difficulties, ethical issues, and significant individual difference in microbial composition (Wos-Oxley et al., 2012). Traditionally, these models require inoculating fecal microbiota from human donors into germ-free mice (Arrieta et al., 2016). However, there are some basic and logical limitations to using germ-free mice as receptors for human microbiota, such as the low immune capacity, the high cost of maintaining germ-free facilities, and the few genotype mice models (Olszak et al., 2012; Cox et al., 2014; Hintze et al., 2014). In order to avoid these restrictions, an alternative HMA-based mice model was developed, using pseudo germ-free mice and a common antibiotic combination of ampicillin, neomycin sulfate, metronidazole, and vancomycin (Ge et al., 2017; Gong et al., 2018). These antibiotics were able to clear the intestinal microbiota, and the pseudo germ-free mice model could be established.

Several groups have successfully established different disease models from pseudo germ-free mice. The Gregory et al. (2015) established HMA mice that are atherosclerosis prone using C57BL/6J *Apoe*^–/^*^–^* pseudo germ-free mice. Similarly, the Ge et al. (2017) transplanted fecal microbiota from constipation patients into pseudo germ-free mice to demonstrate that the pathogenesis of constipation is related to intestinal microorganism. These studies have demonstrated that human bacteria associated with the disease can be transplanted and stabilized in pseudo germ-free mice; however, research of the production mechanism of equol with humanized mice has not been reported, and the humanized mice model of producing equol based on pseudo germ-free mice has not yet been established. The humanized mice of equol production is a model for the transformation of functional actives of intestinal microbiota, and does not represent a model of fecal microbiota transplantation (FMT) for the treatment of diseases. There are no data in literature related to production mechanisms of equol in HMA mice models, which are based on germ-free mice. The equol producing HMA mice model is only valid for functional activities of intestinal microbiota, and not a model for microbiota transplants and treatment. The differences between the two models are that the functional model focuses on the study of biological transformation of substances *in vivo* and the influence of some substances on the composition or metabolism of microbiota (Wang et al., 2018). The disease model focuses on the pathogenesis of the disease and the therapeutic effect of a substance on the disease. The modeling conditions of each functional active substance need to be optimized; therefore, we need to explore new modeling conditions when establishing the HMA mice model for equol production. Previous experiments have obtained 12 equol producers and their fecal microbiota. This study demonstrates that the ability to produce equol in HMA mice could be transferred from the equol producer.

The focus of this study was to establish a humanized mice model of producing equol through pseudo germ-free mice and FMT. Therefore, the study attempted to establish pseudo germ-free mice adopting the reported antibiotic regimen. Sufficiently large quantities of fresh fecal microbiota were transplanted to pseudo germ-free mice. The colonized fecal microbiota of HMA mice were detected by using 16S rRNA gene amplicon sequencing. Finally, the intestinal microbiota composition of HMA mice were compared to the donor and the equol productivity of the HMA mice was determined.

## Materials and Methods

### Selection of Equol Producers

The main objective of our study is to develop a protocol for establishing an HMA model producing equol based on pseudo germ-free mice; therefore, the gender of the subjects is not significant, and both female and male subjects are equally suitable for establishing this model. Since this study was based on the successful colonization of female equol producers in germ-free mice (Bowey et al., 2003; Tamura et al., 2004), the present study chose female volunteers to ensure the success of the model. Fifteen young women (age range 22 ∼ 26; BMI 19.6 ± 1.4) with habits of eating soy products were selected at the South China Agricultural University (Guangzhou, China). None of the participants suffered from any bowel disease. In addition, they had not taken antibiotics or any other drugs for at least 6 months before the beginning of the study. Morning urine samples were collected by the volunteers. Ascorbic acid (0.1%, w/v) was added to urine as a preservative. These samples were divided into two parts, one part of 1 mL volume, and the second aliquot contained the rest. These samples were stored at −80°C until analysis. No human clinical trials were conducted in this study. This study was conducted in agreement with regulations stated in the World Medical Association’s Declaration of Helsinki, and after receiving formal written consent from all subjects. Ethical review and approval were not required for the study on human participants in accordance with the local legislation and institutional requirements.

The main purpose of our article is to establish a protocol for the development of a humanized mice model producing equol based on pseudo germ-free mice. For this purpose, we selected the most suitable equol producer as our donor. A threshold value for the log_10_-transformed urinary equol: daidzein ratio of −1.75 provided a demarcation to define equol producer status because it is independent of isoflavone intake and minimizes interindividual variation in isoflavone pharmacokinetics and differences in analytical methodologies (Setchell and Cole, 2006). The equol producer with the highest value of Log_10_ (equol/daidzein) was first considered as a donor. Then, three fecal samples were taken for *in vitro* experiment of the transformation of daidzein to equol every 3 days to test the capacity of the fecal microbiota to produce equol. One fecal sample from each week was used to detect the similarity of the fecal microbiota composition of the donor through 16S rRNA gene amplification sequencing. Only the equol producer with a similar microbiota composition and the ability to produce equol during colonization was selected as donor.

### High Performance Liquid Chromatography Analyses of Daidzein and Equol in Urine

Urinary samples were prepared as described by Li et al. (2012). Each 1-mL urine sample was centrifuged at 850 × *g* for 15 min at 4°C and then mixed with a 4 mL sodium acetate buffer (0.1 mol/L, pH 5.0), a 20 μL of β-glucuronidase (200 U/mL), and sulfatase (20 U/mL). The mixture was incubated for 2 h at 37°C, followed by extraction using 6 mL of ethyl acetate. The solvent was removed with a rotary evaporator and the dried sample was redissolved in 1 mL of 100% methanol before analysis.

Daidzein and equol concentrations were determined as previously described (Li et al., 2012); a Waters HPLC system, consisting of a Waters 2695 separations module, Waters 2489 fluorescence detector, and Empower-3 software (Waters, Finnigan, CA, United States). A 20 μL sample was injected and separated over an Agilent C_18_ (250 × 4.6 mm column, particle size 5 mm, Shanghai, China). The temperature was set at 30°C and the flow-rate was maintained at 0.8 mL/min. Elution of the compounds was carried out under isocratic flow conditions, with a mobile phase consisting of acetonitrile:methanol:water (20:30:50). Equol was detected at a wavelength of 230 nm and daidzein at 260 nm. Mixed standards of daidzein and equol (Yuanye; Shanghai, China) at five different concentrations (6.25, 12.5, 25, 50, and 100 μg/mL, respectively), were included in each high performance liquid chromatography (HPLC) test and the concentrations determine by triplicate analysis. The concentrations of equol and daidzein were determined using the calibration curves, determined in Origin, ver. 8.6 (OriginLab Corporation, Northampton, MA, United States).

### Animals, Diet and Sample Collection

Six-week-old specific pathogen-free (SPF) C57BL/6J female mice (SCXK 2018-0002) were provided by the Guangdong Medical Laboratory Animal Center (Foshan, China). Mice were divided into two groups, with five mice in each group, and placed in ventilated cages separately under a 12:12 light cycle. Mice were fed with irradiated and autoclaved AIN 93 M diet mouse chow (Guangdong Medical Experimental Animal Center, Foshan, China) and autoclaved water at a constant temperature of 21 ∼ 22°C, and kept at a humidity of 55 ± 5%. The experiment was conducted according to the guidelines of the Experimental Animal Ethics Committee of South China Agricultural University in China (No. 2019028).

**FIGURE 1:**
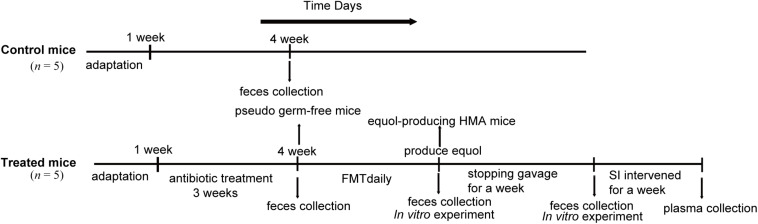
Experimental design indicating the establishment process and sampling times of HMA mice. Two groups of mice received different treatments: control mice and treated mice. The control mice do not require any treatment. The treated mice were established by antibiotics administration 3 weeks and fecal microbiota transplantation (FMT) daily. *In vitro* experiment was performed to check that transplanted microbiota produced equol, and HMA mice were treated with soybean isoflavones (SI) for a week to further determine the success of the model. Fecal samples of control mice were collected at week 4 and treated mice were collected at three different states, pseudo germ-free mice, equol-producing HMA mice, HMA mice after stopping gavage for a week.

The mice were first given a normal diet for 1 week to adapt to the environment before being randomly assigned to two groups of five (Figure 1). One group was used as the control group. The second group received antibiotics administration and FMT and was referred to as treated mice. In order to eliminate the original intestinal microbiota of mice as much as possible, we adopted a 3-week antibiotic treatment regimen. Then, the human fecal suspension were inoculated daily until the fecal microbiota of HMA mice successfully transformed soy isoflavones to equol *in vitro*, which is a direct evidence of the success of equol production in HMA mice model. Finally, feces of HMA mice were collected for 1 week after the suspension was stopped, and the stability of colonization microbiota was tested. The feces from the successful models of pseudo germ-free mice and HMA mice producing equol were collected and immediately frozen at −80°C until further use.

### Establishment of Pseudo Germ-Free Mice and HMA Mice Models

Vancomycin (100 mg/kg) neomycin sulfate (200 mg/kg), metronidazole (200 mg/kg), and ampicillin (200 mg/kg) (Gong et al., 2018) were fed daily to the mice for a period of 3 weeks, in order to create pseudo germ-free mice. All antibiotics were purchased from Macklin (Shanghai, China). For establishing the HMA mice, fresh equol producer feces samples were collected daily (Staley et al., 2017), and 1 g of each sample was diluted in 10 mL of reduced phosphate buffered saline (pretreated in an anaerobic environment for 48 h to reduce oxygenation reduction potential) to make suspensions under anaerobic condition during colonization (Turnbaugh et al., 2009). The pseudo germ-free mice were given 0.2 mL of the fecal suspension daily until the mice were able to produce equol. Then, the fecal suspension was stopped for 1 week, and the feces of HMA mice were collected for the stability detection of colonized microbiota. Finally, the HMA mice were treated with soy isoflavone (100 mg/kg) for 1 week, and the plasma was taken to detect daidzein and equol level, to further determine the success of the model.

### *In vitro* Incubation of Isoflavones With Fecal Microbiota of HMA Mice and Donor

In order to detect the equol producing capacity in HMA mice and donor, this study used the method of extracorporeal conversion of isoflavones from fecal microbiota of HMA mice and donor. Daidzein and equol in the fecal incubation solution with soybean isoflavone were analyzed as follows. Isoflavones (333 μg/mL, Yuanye, Shanghai, China) were dissolved separately in brain heart infusion (BHI) broth medium. The freshly excreted mice (25 ∼ 30 mg) or human feces (∼10 mg) were crushed and added to broth medium with isoflavones substrate, and fermented for 48 h at 37°C under anaerobic condition (Tamura and Saitoh, 2006). One milliliter of the culture medium was mixed with 6 mL of ethyl acetate, and the mixture vortexed for 60 s, and centrifuged at 6000 × *g* for 5 min. After solvent evaporation, methanol dissolution, and filtration, the filtrate was further analyzed by HPLC.

### Analysis of Plasma Isoflavones in HMA Mice

Daidzein and equol in the plasma were analyzed by HPLC after obtaining 200 μL of plasma by centrifugation. Two hundred microliter plasma were mixed with an equal volume of sodium acetate buffer (0.1 mol/L, pH 5.0) containing ascorbic acid (5.7 mmol/L), EDTA (0.27 mmol/L), β-glucuronidase (20 U) and sulfatase (10 U), and incubated at 37°C in a shaking water bath for 2 h, treated with 600 μL of ethyl acetate and vortexed for 30 s, sonicated for 30 s, vortexed again for 30 s, and centrifuged for 15 min at 4°C and 800 × *g* (Tamura and Saitoh, 2006). The supernatant was transferred to a rotary evaporator for complete solvent removal. The sample was then resuspended in methanol to the original volume.

### Calculation of Bacterial Load in Mice by Quantitative Real-Time PCR

The DNA was extracted from the fecal samples of mice according to the procedure described by Bitzer et al. (2016) and Wang et al. (2018). The extracted genomic DNA was used for Q-PCR of the V4 hypervariable region of the bacterial 16S gene. The primers (289 bp) were applied, 5′-AGGCAGCAGTGGGGAAT-3′; 5′-GCCAGCAGCCGCGGTAA-3′ (Manichanh et al., 2010). The Q-PCR calibration curve was established in accordance with the methods of Manichanh et al. (2010). In order to infer the number of bacteria in each sample, a continuous dilution of plasmid was amplified (DNA copy number ranging from 122 to 1.22 × 10^7^).

The CFX96 Real-time PCR System (Bio-Rad Laboratories, Hercules, CA, United States) was used to amplify the extracted DNA. The final volume of all amplifications was 25 μL, containing 12.5 μL of the 2 × AceQ^®^ Universal SYBR qPCR Master Mix (Vazyme, Nanjing, China), 1 μL of each primer (10 μM), and 5 μL of template DNA. The protocol for amplification was as follows: an initial cycle at 95°C for 5 min, followed by 40 cycles at 95°C for 10 s, and 30 s at 60°C. In order to check specificity, the melting curve (TM) analysis was carried out over a temperature range from 60 to 95°C, and a temperature ramp rate of 0.2°C per second. The average values were determined from three parallel reactions.

### Illumina MiSeq DNA Sequencing and Bioinformatics

Fecal samples (50 mg) from all mice and donor were sent to the Gene *Denovo* Biotechnology Company (Guangzhou, China) for 16S rRNA gene amplification sequencing. The DNA of fecal bacteria was extracted by using a DNA extraction kit. Its integrity and size were confirmed by 1% agarose gel electrophoresis. The V3 and V4 domains of biological 16S rRNA gene were quantified by PCR with 341 F and 806 R primers. The amplified fragment was purified and sequenced on the platform of Hiseq 2500 PE 250 platform. The software “Quantitative Insights into Microbial Ecology” (QIIME) was used to analyze the sequence data.

The operational taxonomic units (OTUs) were assigned for the 16S rRNA gene sequences with a threshold of 97% for paired recognition, and were classified using ribosome database project (RDP) classifier 2.0.1. The α diversity estimates were calculated by the “Chao richness” and “Shannon diversity” index. Principal coordinate analysis (PCoA) was used to study the differences among gut microbial communities of different groups.

### Statistical Analysis

Origin 8.6 (OriginLab Corporation, Northampton, MA, United States) was used for all analyses and preparation of graphs. Continuous data were presented as the means ± standard error of mean (SEM). Analysis of variance (one-way ANOVA) or one-sample *t*-test were used to analyze the significance of the data. Significance was accepted at *p* < 0.05. If not otherwise specified, statistical significance was indicated as follows: ^∗^*p* < 0.05; ^∗∗^*p* < 0.01; ^∗∗∗^*p* < 0.001.

### Availability of Data and Materials

The dataset supporting the conclusion of this article is available in the National Center for Biotechnology Information (NCBI) Sequence Read Archive (SRA) repository, BioProject ID PRJNA597182.

## Results

### Identification and Characteristics of High Equol-Producer

Equol concentrations were determined by HPLC analysis (Supplementary Figure S1), adopting a threshold of: Log_10_ (equol/daidzein) ≥ −1.75 for the equol producer status, to ensure the selection of volunteers with high levels of equol (Setchell and Cole, 2006). The results showed that ***12*** of the ***15*** subjects were equol producers, whereas the other three did not produce any equol (Table 1). For equol producers, the log_10_ (equol/daidzein) indicators ranged from −0.76 to 0.63, and the highest value (0.63) belongs to subject 4, suggesting that the intestinal microbiota related to glycoside hydrolase and equol production were active in this equol producer, and was chosen as the donor.

**TABLE 1:** Daidzein and equol concentration in urine samples among equol producer and non-producer women (μmol/L).

Urinary isoflavone	Subject number	Daidzein	Equol	Log_10_ (equol/daidzein)
Equol-producers	1	6.19	17.08	0.44
	2	9.64	28.41	0.47
	3	9.19	13.99	0.18
	4	5.57	23.97	0.63
	5	14.38	27.52	0.28
	6	32.75	37.08	0.05
	7	12.73	29.85	0.37
	8	7.06	21.49	0.48
	9	63.15	29.88	–0.32
	10	74.88	29.71	–0.40
	11	80.46	28.56	–0.45
	12	80.46	14.02	–0.76
Non-equol producers	13	20.02	0.00	ND
	14	24.91	0.00	ND
	15	34.35	0.00	ND

*ND, not detected.*

**FIGURE 2:**
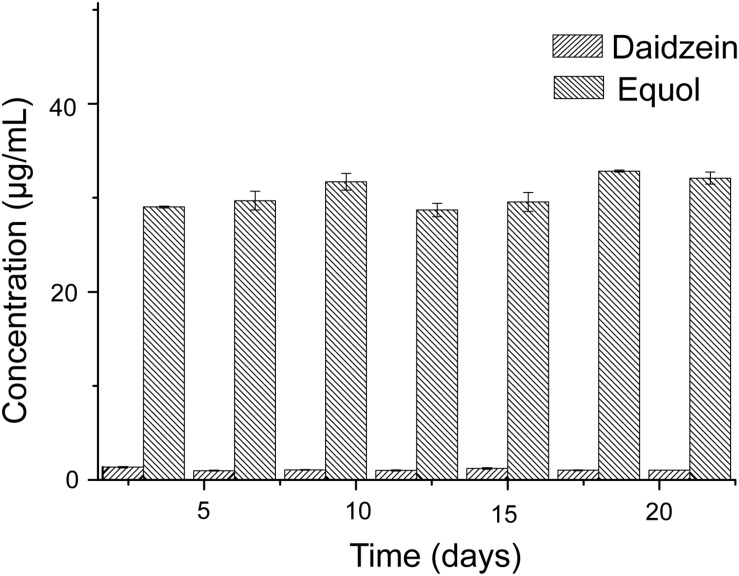
Conversion of daidzein with fecal microbiota of donor *in vitro*.

The experiment of the transformation of daidzein to equol was conducted *in vitro*, to test the capacity of the donor microbiota to produce equol during the inoculation period. Literature data have shown that some active bacteria can produce equol in feces of equol producers and their fecal microbiota can produce equol *in vitro* in fecal incubation solution with isoflavones or daidzein (Guadamuro et al., 2017). Our results showed that the fecal microbiota of the donor could convert 50 μg/mL of daidzein into 30.52 ± 1.53 μg/mL of equol within 48 h and repeatedly over a time frame of 3 weeks (Figure 2), indicating that equol production phenotype proved to be stable over the study period. The above analysis showed that the external interference has little effect on the ability of the donor microbiota to produce equol.

**FIGURE 3:**
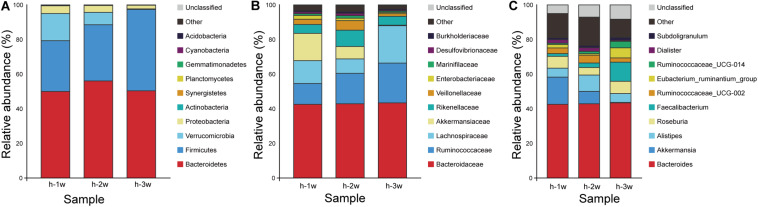
Microbiota composition of donor at three time points: week 1 (1 w), week 2 (2 w), and week 3 (3 w). **(A)** Phylum level. **(B)** Family level. **(C)** Genus level. Group h, donor.

The stability of the composition of the donor microbiota was determined by randomly selecting a fecal sample for 16S rRNA gene amplification sequencing every week during the 3 weeks experiment period. Taxonomy data obtained from 3 weeks of fecal samples of the donor showed that members of Bacteroidetes and Firmicutes had high relative abundance, while Verrucomicrobia and Proteobacteria possessed low relative abundance (Figure 3A). In all samples, Bacteroidetes were mainly represented by the families *Bacteroidaceae* and *Rikenellaceae* (Figure 3B) and the genus *Bacteroides* and *Alistipes* (Figure 3C; Hamilton et al., 2013). Compared with Bacteroidetes, Firmicutes in donor samples were composed of more diverse families, such as *Ruminococcaceae*, *Lachnospiraceae*, and *Veillonellaceae* (Figure 3B). The data of the microbiota composition of donor are similar at the phylum level, but the relative abundance of family and genus levels fluctuated during 3 weeks, which had little effect on the FMT.

### The Equol Producing Capacity of HMA Mice

In our experiment, we were able to detect equol in the fecal incubation solution of HMA mice after 21st days of inoculation, indicating that our model for equol production in HMA mice was successful. We tested the intestinal microbiota composition of the HMA mice for 28th days to observe the stability of the colonized microbiota and the capacity of producing equol. In order to demonstrate the capacity of equol production in HMA mice, the following two protocols were used in this study: the first was *in vitro* incubation of isoflavones with fecal microbiota of HMA mice. The second protocol consisted of feeding soy isoflavones to HMA mice for 1 week, and measure equol concentrations in their plasma.

**FIGURE 4:**
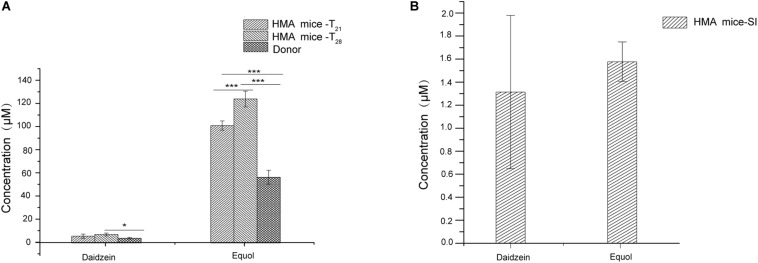
The capacity of producing equol in HMA mice. **(A)** Concentration of daidzein and equol in *in vitro* incubaction of fecal microbiota of HMA mice with isoflavones. **(B)** Plasma concentrations of daidzein and equol of analysis in HMA mice. *p-value < 0.05; ***p-value < 0.001.

Previous studies have shown that fecal microbiota of women equol producer could generate equol *in vitro* during fecal fermentations with isoflavones. These findings might indicate that certain active bacterial types in feces could produce equol (Decroos et al., 2006; Tamura et al., 2011; Guadamuro et al., 2017). The presence of equol producer phenotypes during isoflavone supplementation might provide evidence for the production of equol in HMA mice model through *in vitro* experiments. For *in vitro* experiments, both daidzein and equol were successfully detected in isoflavone incubation solution. No significant differences were observed in daidzein concentration between HMA mice – T_21_ and T_28_. However, daidzein concentrations in HMA mice – T_28_ were significantly higher than the donor (*p* < 0.05). In contrast, equol concentrations were much higher in the HMA mice – T_28_ than in the HMA mice – T_21_ and donor (*p* < 0.001) (Figure 4A). For *in vivo* experiments, daidzein and equol were detected in the plasma of HMA mice, which received isoflavones in our study (Figure 4B). The *in vitro* and *in vivo* experiment analyses indicated that HMA mice were capable of producing equol and HMA mice model producing equol were established.

### Alpha Diversity Indices of Mice and Donor

**FIGURE 5:**
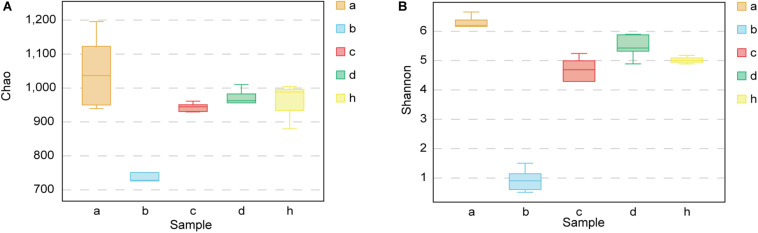
Chao richness and Shannon diversity index for SPF mice, pseudo germ-free mice, HMA mice and donor. **(A)** Chao richness. **(B)** Shannon diversity index. Group a (*n* = 5), SPF mice without antibiotics; Group b (*n* = 5), pseudo germ-free mice; Group c (*n* = 5), HMA mice – T_21_; Group d (*n* = 5), HMA mice – T_28_; Group h, donor.

The Chao richness estimations and Shannon diversity index for each sample are shown in Figure 5. After treating mice with antibiotics, the bacterial composition changed significantly. The Chao index decreased from 1047.79 ± 110.56 to 745.05 ± 81.32 (*p* < 0.01) (Figure 5A), and Shannon index decreased from 6.23 ± 0.31 to 0.92 ± 0.40 (*p* < 0.001) (Figure 5B). The diversity of fecal microbiota of HMA mice – T_21_ was not significantly lower than that of HMA mice – T_28_ [Chao richness estimations of 942.26 ± 13.41 (HMA mice – T_21_) versus 949.10 ± 65.14 (HMA mice – T_28_) (Figure 5A); Shannon index of 4.41 ± 0.91 (HMA mice – T_21_) versus 5.47 ± 0.42 (HMA mice – T_28_) (Figure 5B)]. The Chao index of donor was 956.20 ± 67.69 and Shannon index was 5.01 ± 0.14. No significant differences were observed in the diversity indices between the HMA mice – T_21_, T_28_ and donor. These results showed that the diversity of HMA mice recovered to reach almost the same level as that of the donor after FMT. The diversity of HMA mice microbial communities did not differ as a function of time.

### The Principle Coordinate Analysis of Mice and Donor

**FIGURE 6:**
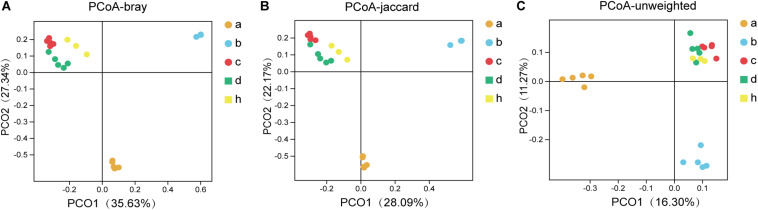
The principal coordinates analysis (PCoA) for SPF mice, pseudo germ-free mice, HMA mice and donor. **(A)** PCoA – bray (separation is based on species availability and species abundance). **(B)** PCoA – jaccard (separation is based on the differences in the microbiota structure of the species). **(C)** PCoA – unweighted (separation is based on phylotype presence). Group a (*n* = 5), SPF mice without antibiotics; Group b (*n* = 5), pseudo germ-free mice; Group c (*n* = 5), HMA mice – T_21_; Group d (*n* = 5), HMA mice – T_28_; Group h, donor.

The “Microbiota Array” method of PCoA was used to analyze the phylogeny type data and identify the microbial communities of all samples (Kelly et al., 2012). The correlation analysis emphasizes the concentration differences of the various groups, i.e., SPF mice without antibiotics, pseudo germ-free mice, HMA mice – T_21_ and T_28_, and donor (Figure 6). Consistent with the above analysis, there were significant differences in the structure of community phylogeny types between SPF mice and pseudo germ-free mice samples along the *x*-axis (Figure 6). The compound abundance in HMA mice – T_21_, T_28_ samples, and donor samples supports the assumption that the intestinal microbial community structure of HMA mice originated from the fecal microbiota of the donor (Figure 6).

### Establishment of HMA Mice Model Producing Equol

**FIGURE 7:**
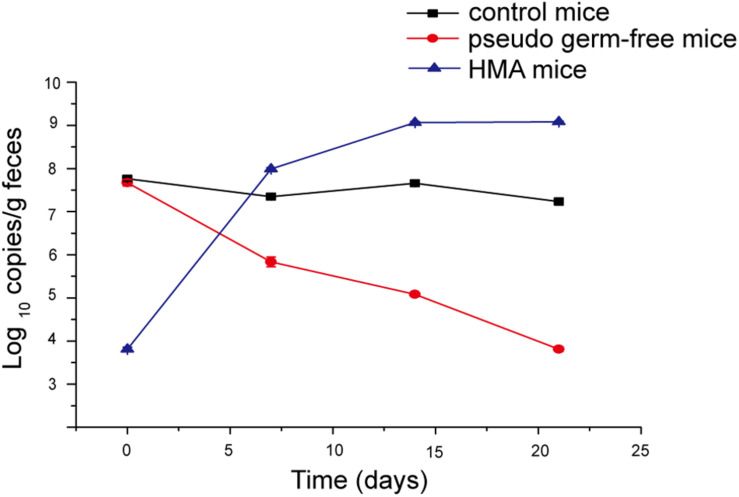
Bacterial quantification assessed by real-time PCR of the 16S gene at four time points: day 0, day 7, day 14, and day 21.

Pseudo germ-free mice were treated by broad-spectrum antibiotics for 3 weeks. As expected, the number of microorganisms in fecal samples of pseudo germ-free mice decreased from 10^7^ to 10^3^, and the bacterial load of SPF mice without antibiotics fluctuated within 10^7^ orders of magnitude (Figure 7). The intestinal microbial community of pseudo germ free mice declined significantly relative to the SPF mice without antibiotics. The analyses indicated that the number of bacteria in pseudo germ-free mice decreased to a very low level, which was very beneficial for the colonization of exogenous bacteria. After the pseudo germ-free mice were inoculated with the donor’s microbiota, the bacterial load of HMA mice increased to 10^9^ (Figure 7), indicating that the microbiota of HMA mice had recovered.

**FIGURE 8:**
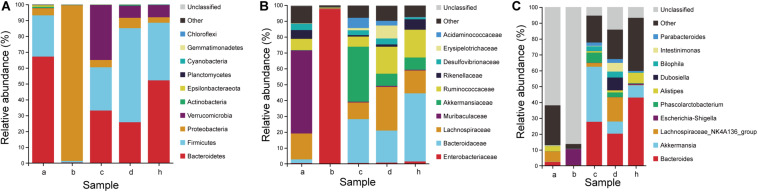
Microbiota composition of SPF mice, pseudo germ-free mice, HMA mice and donor. **(A)** Phylum level. **(B)** Family level. **(C)** Genus level. Group a (*n* = 5), SPF mice without antibiotics; Group b (*n* = 5), pseudo germ-free mice; Group c (*n* = 5), HMA mice – T_21_; Group d (*n* = 5), HMA mice – T_28_; Group h, donor.

The antibiotic clearance of the gut microbiota in mice were studied (Figure 8). After the antibiotic treatment, the change of species composition was mainly due to expansion of Proteobacteria (98.10%) and extinction of Bacteroidetes (0.39%) and Firmicutes (0.95%) (Figure 8A). The dominant family in mice hanged from *Lachnospiraceae* (16.34%), *Muribaculaceae* (52.40%) and *Ruminococcaceae* (7.14%) to *Enterobacteriaceae* (97.52%) (Figure 8B). The dominant genera in mice changed from the *Lachnospiraceae_NK4A136_group* (6.50%) and *Alistipes* (3.19%) to *Escherichia-Shigella* (10.19%) (Figure 8C). Therefore, the proportion of dominant phylum and family decreased significantly and the gap increased, resulting in the overgrowth of Proteobacteria and *Enterobacteriaceae*. Due to the depletion of most intestinal microbiota, humanized mice models could be based on pseudo germ-free mice under the limited germ-free facilities.

To determine which species were transferred from the donor to the HMA mice, the fecal microbiota composition of HMA mice – T_21_ in comparison with the human-donor were analyzed. The taxonomy data from HMA mice revealed similar taxa to the donor sample, but the relative abundance varied considerably. These data indicated that transference of fecal microbiota from donor to HMA mice was particularly efficient for Bacteroidetes, Firmicutes, Verrucomicrobia, and Proteobacteria (Figure 8A). In the establishment of Bacteroidetes, the *Bacteroidaceae* were also colonized successfully in mice, which was established in mouse systems at an approximate abundance of 27.70% (Figure 8B). In the establishment of Firmicutes, *Ruminococcaceae* and *Lachnospiraceae* (Cluster IV *Clostridia* members) were reported to contribute 6.4 and 10.6% of the total community (Figure 8B). The above analysis indicated that the dominant microbiota of the equol producer was colonized successfully by HMA mice.

To examine the persistence of HMA colonized microbiota, the fecal microbiota composition of HMA mice – T_21_ and T_28_ were analyzed. The HMA mice – T_21_ and T_28_ samples have similar taxonomy data. In Phylum level, the relative abundance of Bacteroidetes were 33.10 and 25.74%, and the relative abundance of Firmicutes were 27.26 and 59.28% in HMA mice – T_21_ and T_28_, respectively. In Family level, the relative abundance of *Bacteroidaceae* accounted for 27.70 and 20.22%, and *Ruminococcaceae* were reported to contribute 6.39 and 17.04%, and *Lachnospiraceae* contributed 10.46 and 27.57% of the total HMA mice – T_21_ and T_28_ community (Figure 8B). In genus level, the relative abundance of *Bacteroides* was established in HMA mice – T_21_ and T_28_ with an approximate abundance of 27.70 and 20.22%, and *Akkermansia* accounted for 34.66 and 7.66% (Figure 8C). The analysis indicated that the microbiota of HMA mice were stable and did not disappear after 3 weeks.

The relative abundance of the *Coriobacteriaceae* (capable of synthesizing equol) was compared in the human and HMA mice. Even though the donor samples have a lower abundance of *Coriobacteriaceae* (∼0.05%), the HMA mice – T_21_ and T_28_ have a slightly higher abundance following transplantation (∼0.16 and 0.06%) (Table 2). It should be emphasized that the relative abundance of *Collinsella* accounted for 0.03% of bacteria sequences in equol producer and 0.01% of bacteria sequences in HMA mice – T_21_ and T_28_. At the genus level, the genera (*Alistipes*, *Roseburi*a, *Oscillibacter*, *Blautia*, *Faecalibacterium*, and *Bifidobacterium*) found in both donor samples and HMA mice might be associated with equol production.

**TABLE 2:** Family and genera related to soybean isoflavone metabolism in relative abundance (% sequences) between HMA mice – T_21_, T_28_ and donor.

Family and genera	HMA mice – T_21_ (*n* = 5)	HMA mice – T_28_ (*n* = 5)	Donor
*Coriobacteriaceae*	0.16 ± 0.09	0.06 ± 0.01	0.05 ± 0.02
*Collinsella*	0.01 ± 0.00	0.01 ± 0.00^***^	0.03 ± 0.01
*Alistipes*	0.84 ± 0.10^***^	2.85 ± 0.22^***^	6.33 ± 1.33
*Roseburia*	0.05 ± 0.00^***^	0.08 ± 0.01^***^	6.00 ± 1.00
*Oscillibacter*	0.05 ± 0.00^∗∗^	8.35 ± 1.06^∗^	0.09 ± 0.00
*Blautia*	0.64 ± 0.36	0.56 ± 0.37	0.13 ± 0.03
*Faecalibacterium*	0.01 ± 0.00^***^	0.02 ± 0.00^***^	5.33 ± 2.85
*Bifidobacterium*	0.01 ± 0.00^∗∗^	0.42 ± 0.23	0.03 ± 0.01

*^∗^p-value < 0.05; ^∗∗^p-value < 0.01; ^∗∗∗^p-value < 0.001 compared to donor.*

## Discussion

These results presented above support the feasibility of transplanting the microbiota of equol producer to pseudo germ-free mice via fecal microbiota transfer. In this work, we were able to establish an HMA mice model for equol production, based on pseudo germ-free mice. Previous research studies have confirmed that the ability to produce equol could be transferred to germ-free rats or mice through fecal samples from equol producers (Bowey et al., 2003; Tamura et al., 2004; Tamura and Saitoh, 2006). However, there are some unavoidable limitations in using germ-free mice as receptors. Due to the lack of intestinal microbiota, germ-free mice have low immune and negative metabolic capacities. In addition, the development and maintenance of germ-free equipment require expensive resources, and few germ-free mouse genotypes are available for selection (Olszak et al., 2012; Cox et al., 2014; Hintze et al., 2014). In our model, the intestinal immune system of pseudo germ-free mice has already undergone maturation during development, which can overcome the low immunity of germ-free mice (Staley et al., 2017). Further, the mice are housed in microisolator cages supplied with high-efficiency particulate air filter, and do not require expensive sterile facilities and specialized personnel (Beura et al., 2016). Notably, like the HMA mice made from germ-free mice, the current model established from pseudo germ-free mice also produced equol. The result indicated that the HMA mice model for equol production, based on pseudo germ-free mice, could be an alternative and powerful model for carrying out research in the mechanism of conversion of isoflavones into equol.

The HMA model development process and the effect of fecal microbial transplantation consisted of several steps: first, we analyzed the elimination effects of gut microbiota produced by antibiotic treatment. The broad-spectrum antibiotics (vancomycin, neomycin sulfate, metronidazole, and ampicillin) were administered intragastrically once daily for 3 weeks. After 3 weeks of treatment, the gut microbiota composition and species richness of pseudo germ-free mice was significantly reduced, and PCoA analysis showed that the microbiome of pseudo germ-free mice clustered far away from the SPF mice. The results showed that the intestinal tract was still not completely sterile even after long-term exposure to the same antibiotics. This type of phenomenon has been reported by several other research groups (Ichinohe et al., 2011; Dapito et al., 2012; He et al., 2015; Schuijt et al., 2016). One likely explanation is that low numbers of remaining microorganisms are required to protect the niches in the gastrointestinal tract throughout antibiotic treatment (Macfarlane and Dillon, 2007). Furthermore, the combination of antibiotics presents a selective pressure on the intestinal microbiota of pseudo germ-free mice, which resulted in an increase in the relative abundance of aerotolerant species and a decreasing number of obligate anaerobic species, as already pointed out in the results section. However, there are slight differences within the remaining species and the degree of reduction in species richness between our research and other studies (Ericsson et al., 2017; Ge et al., 2017). Such differences may be a result of the duration of administration and the combination of antibiotics. First, it should be recognized that long durations of feeding antibiotics are more conducive to the elimination of intestinal microbiota. Second, multiple antibiotic courses destroyed the common features within the local mouse community (Staley et al., 2017). In short, the present antibiotic regimen is an alternative in the establishment of pseudo germ-free mice.

Next, we characterized the colonization potential of the donor’s fecal sample into pseudo germ-free mice. In the donor gut microbial community, phylum Bacteroides were dominant, accounting for 50–56% of the bacterial sequence. The relative abundance of Firmicutes were secondary, accounting for 29–47% of the bacterial sequence. Firmicutes contained the most diverse component of phylotypes-*Clostridia*, accounting for 82–96% of the Firmicutes sequence. These results are comparable to those reported for the detection of microbiome of healthy individuals (Khoruts et al., 2010). For the colonized microbiota of HMA mice, the dominant phylum (Bacteroidetes, Firmicutes, and Verrucomicrobia) were successfully transferred from human donor. The dominant family (*Bacteroidaceae*, *Akkermansiaceae*, *Lachnospiraceae*, and *Ruminococcaceae*) also transferred successfully from the donor to HMA mice. Turnbaugh et al. (2009) also observed similar enrichment of Bacteroides after inoculating fecal microbiota into germ-free mice. Kumar et al. (2016) also found that the proportion of *Bacteroidaceae* and *Lachnospiraceae* of the gnotobiotic mice transplanted with the donor fecal microbiota increased to levels similar to the donor samples. The above analysis indicated that the overall degree of fecal microbiota engraftment for equol producers in pseudo germ-free mice was in the same range as for germ-free mice or gnotobiotic mice, due to the long-term and substantial transplantation of human microbiota after antibiotic treatment. Although substantial transplantation required a dense feeding schedule (Hintze et al., 2014), it will make the engraftment target simple and clear to use for the production of equol in the current experiment.

Finally, we analyzed the equol producing bacteria characteristic for HMA mice and donor. Our study found *Coriobacteriaceae* and *Collinsella* in HMA mice and donor samples. *Coriobacteriaceae* has been described as containing the potential to synthesize equol, but *Collinsella* has not been reported to provide substantial evidence to support its ability for equol production, even if an increase in the abundance of the genus after treatment with isoflavones was observed (Maruo et al., 2008; Nakatsu et al., 2014). Although several strains of the family *Coriobacteriaceae* have been found to produce equol (Yokoyama and Suzuki, 2008; Matthies et al., 2009), more information is required. The other genera (*Alistipes*, *Roseburia*, *Oscillibacter*, *Blautia*, *Faecalibacterium*, and *Bifidobacterium*) were also transplanted successfully, which have been reported to be involved in the transformation process from soybean isoflavones to equol conversion (Guadamuro et al., 2017, 2019; Iino et al., 2019). The result indicated that the colonized fecal microbiota had the potential to convert isoflavones into equol.

## Conclusion

The study established a new humanized mice model for equol production through pseudo germ-free mice, and *in vitro* and *in vivo* experiments demonstrated its capacity to produce equol. Future work is still needed to investigate the longer term and intergenerational durability of engraftment. However, the model has the potential for studying the human microbiota associated with equol. The humanized mice model for equol production can be used for *in vivo* intervention of soybean isoflavone, which is helpful to improve and supplement the mechanism of soybean isoflavone conversion to equol under the action of intestinal microbiota. It can also be used to study the effect of some active substances on the equol-producing bacteria, and the associated mechanisms, i.e., enhancement or inhibition of the absorption and metabolism of equol. The platform is economical and broadly available, and we anticipate it will prove complementary to current models relying on germ-free mice.

## Data Availability Statement

The datasets generated for this study can be found in the NCBI SRA, https://www.ncbi.nlm.nih.gov/sra/PRJNA597182.

## Ethics Statement

The animal study was reviewed and approved by the Experimental Animal Ethics Committee of South China Agricultural University. Written informed consent was obtained from the individual(s) for the publication of any potentially identifiable images or data included in this manuscript.

## Author Contributions

LW, WL, and LZ conceived the trial. LW and WL designed the study and primary responsibility for final content. WL, XF, and QZ conducted this research. ZL, JW, and JZ analyzed the data. HL and YG prepared the manuscript. All authors read and approved the final manuscript.

## Conflict of Interest

The authors declare that the research was conducted in the absence of any commercial or financial relationships that could be construed as a potential conflict of interest.
