# Supplementary material for: Colonization Potential to Reconstitute a Microbe Community in Pseudo Germ-Free Mice After Fecal Microbe Transplant From Equol Producer
Source: Front Microbiol. 2020 Jun 5;11:1221. doi: 10.3389/fmicb.2020.01221 (PMC7291926; doi:10.3389/fmicb.2020.01221)
Supplement: Supplementary file 1 [file Data_Sheet_1.docx]

Supplementary Material

## Supplementary Figures


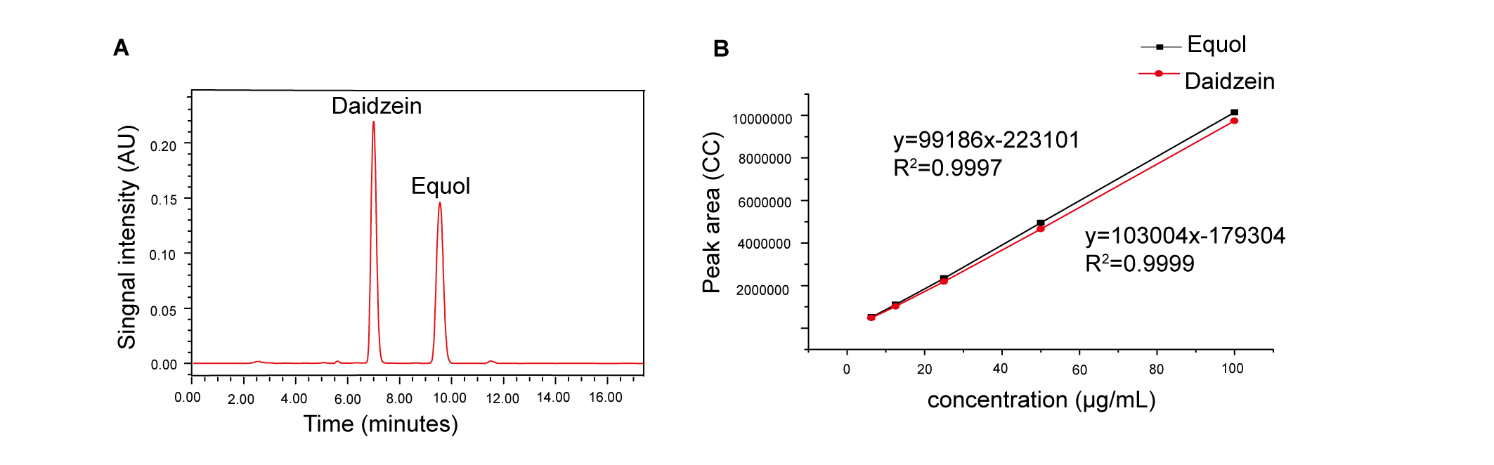


**Supplementary Figure S1 |** Detection of daidzein and equol in urine samples. (A) The standards of daidzein and equol at 25 μg/mL. (B) The relationship between concentrations and peak area in daidzein and equol.
